# How long does a hip replacement last? A systematic review and meta-analysis of case series and national registry reports with more than 15 years of follow-up

**DOI:** 10.1016/S0140-6736(18)31665-9

**Published:** 2019-02-16

**Authors:** Jonathan T Evans, Jonathan P Evans, Robert W Walker, Ashley W Blom, Michael R Whitehouse, Adrian Sayers

**Affiliations:** a Musculoskeletal Research Unit, Translational Health Sciences, Bristol Medical School, Southmead Hospital, Bristol, UK; b Health and Policy Research Group, University of Exeter, Exeter, UK; c Department of Trauma and Orthopaedics, Derriford Hospital, Plymouth, UK; d National Institute for Health Research Bristol Biomedical Research Centre, University Hospitals, Bristol NHS Foundation Trust, University of Bristol, Bristol, UK

## Abstract

**Background:**

Total hip replacement is a common and highly effective operation. All hip replacements would eventually fail if in situ long enough and it is important that patients understand when this might happen. We aimed to answer the question: how long does a hip replacement last?

**Methods:**

We did a systematic review and meta-analysis with a search of MEDLINE and Embase from the start of records to Sept 12, 2017. We included articles reporting 15-year survival of primary, conventional total hip replacement constructs in patients with osteoarthritis. We extracted survival and implant data and used all-cause construct survival as the primary outcome. We also reviewed reports of national joint replacement registries, and extracted data for a separate analysis. In the meta-analyses, we weighted each series and calculated a pooled survival estimate for each source of data. This study was registered with PROSPERO (CRD42018085642).

**Findings:**

We identified 140 eligible articles reporting 150 series, and included 44 of these series (13 212 total hip placements). National joint replacement registries from Australia and Finland provided data for 92 series (215 676 total hip replacements). The 25-year pooled survival of hip replacements from case series was 77·6% (95% CI 76·0–79·2) and from joint replacement registries was 57·9% (95% CI 57·1–58·7).

**Interpretation:**

Assuming that estimates from national registries are less likely to be biased, patients and surgeons can expect a hip replacement to last 25 years in around 58% of patients.

**Funding:**

National Institute for Health Research, National Joint Registry for England, Wales, Northern Ireland and Isle of Man, and The Royal College of Surgeons of England.

## Introduction

Total hip replacement is one of the most common and effective forms of surgery, resulting in generally excellent outcomes.^1^ In an immortal cohort, all hip replacements will eventually fail because of processes such as infection, fracture, or a combination of normal tribological and biological processes, such as loosening and wear.

To counsel patients accurately and appropriately, benchmark treatment strategies, plan health-care provision, and for medicolegal purposes, it is important to know how long a total hip replacement might last. Life expectancy is rising and thus the long-term survivorship of the total hip replacement construct is increasingly relevant. The ultimate aim, that all hip replacements provide normal pain-free function for the rest of the recipients lives, has not been achieved. In the UK, the National Institute of Health and Care Excellence set a benchmark in 2014, that individual components making up a total hip replacement are only recommended for people with end-stage arthritis, if they have 10-year revision rates of 5% or lower.^2^ The Orthopaedic Data Evaluation Panel assists in the implementation of this guidance, producing summary 10-year revision rates for individual prostheses using multiple data sources. At present, it does not publish revision rates for hip replacements, but only constituent parts.

Patients who undergo hip replacement surgery are at risk of revision surgery. The commonest reasons for revision are infection, wear, loosening, dislocation, persistent pain, and fracture. Revision surgery is not as effective as primary surgery for relieving pain and improving function and is more expensive.^3^, ^4^ Furthermore, revision hip replacements fail much earlier than do primaries, necessitating further revisions.^5^

The typical patient who had a hip replacement in the UK in 2016 was 69·8 years old if female or 67·6 years old if male, and had a BMI of 28·8. 90% of hip replacements were done for osteoarthritis and 60% of recipients were female.^6^ Similar demographics are reported by the national registries in Scandinavia, Australia, and the Netherlands.^7^, ^8^, ^9^

We wished to answer a question that is posed to us by our patients many times per day: how long does a hip replacement last?

**Research in context**

**Evidence before this study**

Survival of hip replacements has often been reported in case series, some of which extend to 40 years. We identified 140 articles reporting case series through a search of MEDLINE and Embase (from start of records to Sept 12, 2017) analysing single hip replacement constructs, implanted for osteoarthritis. These articles report estimates of survival at 15 years ranging from 48·0% to 93·2%. However, individual case series are prone to bias and reporting has been highly heterogeneous. Joint replacement registries provide more generalisable data but have only existed since 1975 and have limits in terms of the variables collected and interpretation. Survival of a hip replacement is dependent on the implants used to create a construct and on patient characteristics. A pooled estimate of 20-year survival of 85·0% (95% CI 83·2–86·6) using the UK Clinical Practice Research Datalink database was published in 2017. No study to date has attempted to provide a pooled survival calculation for hip replacements 25 years after surgery.

**Added value of this study**

Our results provide the first survival estimate, drawn from multiple sources, for hip replacements at 25 years. This study benefits from an inclusive design, a-priori inclusion criteria, and a realistic interpretation of survivorship. We estimate that roughly 58% of hip replacements will last 25 years.

**Implications of all the available evidence**

Estimates of the survival of hip replacements will be of use to patients, those providing and commissioning health-care services, and those needing an estimate for medicolegal purposes.

## Methods

### Study design and data sources

We did a systematic review and meta-analysis of case series and cohort studies reporting survival outcomes of total hip replacements. We did a second meta-analysis of national joint replacement registries with more than 15 years of follow-up.

To collect data for the analysis of case series, we systematically searched for case series and cohort studies in English reporting survival outcomes of total hip replacements in MEDLINE and Embase from commencement to Sept 12, 2017. The search of MEDLINE used keywords relating to total hip replacement, survival, and MeSH terms (appendix p 1). The search strategy for Embase was the same. We manually screened the bibliographies of all full-text articles matching our criteria, as well as review articles, for additional citations.

We included studies if they involved predominantly unselected patients or patients undergoing total hip replacement for osteoarthritis. We required the reporting of survival of a specific implant, brand, or construct with a mean or median follow-up of greater than 15 years. We excluded articles reviewing specifically complex primary total hip replacement, revision, or hip resurfacing, because these procedures have different survivorship. We also excluded conference abstracts because they were unlikely to contain enough information. Some articles reported implant construct survivorship from registry data, we excluded these studies to avoid duplication with our separate analysis of joint registries. We retrieved systematic reviews and searched their bibliographies but we did not include pooled data from the reviews themselves to avoid duplication.

For the analysis of data from national joint replacement registries, we assessed the six registries that had greater than 15 years of follow-up for total hip replacement at the time of data collection in December, 2017: Australia, Denmark, Finland, New Zealand, Norway, and Sweden. We reviewed the websites and most recent annual reports of these registries for data on conventional, stemmed, total hip replacement constructs. These national registries collect data on all patients undergoing total hip replacement from both public and private hospitals and their aim is to include all total hip replacements in their cohort.

### Abstract screening and data extraction

We screened the abstracts of journal articles using Rayyan,^10^ by three reviewers (JTE, RWW, JPE) and in cases of disagreement, were included for review. Either JTE or JPE and RWW together extracted data using a standardised proforma. We recorded, when available, data for publication date, implant, fixation, number of total hip replacements, age, sex, indication, loss to follow-up, and summary survivorship estimates (including confidence intervals) at all timepoints reported, as well as data for quality assessment. We did not abstract data from figures to prevent potential inaccuracy. Any discrepancy of data extraction was rectified by review of the full text by all three reviewers. There were no cases of disagreement after this.

### Statistical analysis

Our primary exposure was the total hip replacement construct and all-cause revision of any part of the construct was our endpoint. We did the statistical analysis with Stata (version 15). We pooled survival estimates, assuming that survivorship approximated risk, with meta-analysis weighting each series on the overall pooled estimate according to its standard error (calculated from published confidence intervals).

We assessed study quality using the non-summative four-point system (consecutive cases, multicentre, less than 20% loss to follow-up, and use of multivariable analysis) developed by Wylde and colleagues.^11^ We used this system rather than the summative MINORS score^12^ because of the high loss to follow-up in joint replacement case series and because some of the scoring criteria were not relevant to joint replacement.

This study was registered with PROSPERO (CRD42018085642).

### Role of the funding source

The funder of the study had no role in study design, data collection, data analysis, data interpretation, or writing of the report. The corresponding author had full access to all the data in the study and had final responsibility for the decision to submit for publication.

## Results

**Figure 1:**
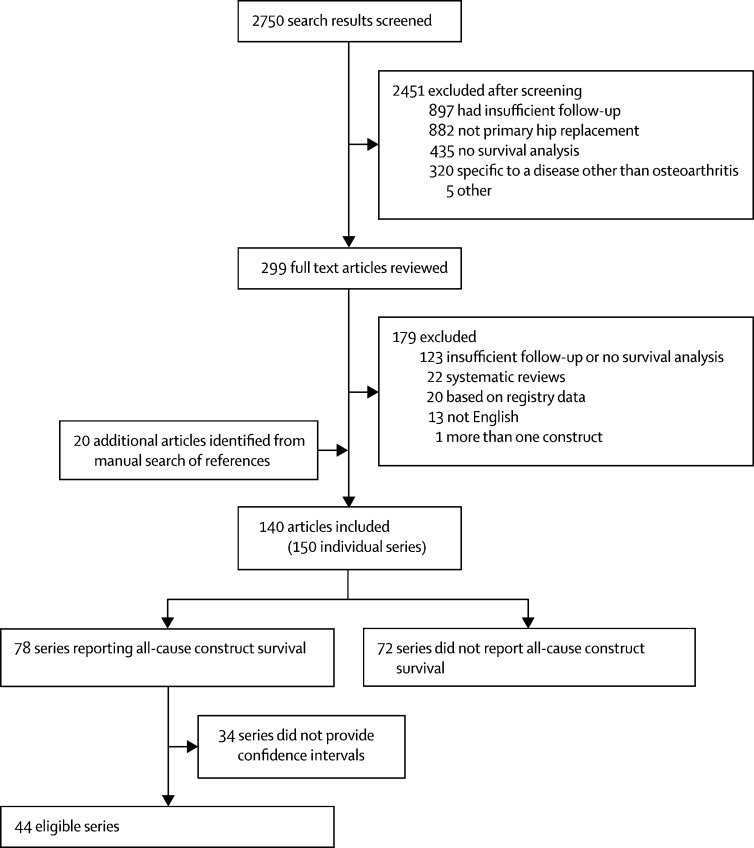
Article selection

Our search produced 4195 references. We removed 1445 duplicates, leaving 2750 reports for screening. After screening, 299 full texts were left for review with 20 additional citations identified by a manual search of references and reviews (appendix p 2). We excluded 13 articles not in English.^13^, ^14^, ^15^, ^16^, ^17^, ^18^, ^19^, ^20^, ^21^, ^22^, ^23^, ^24^, ^25^ Following a review of full texts, 140 journal articles reporting 150 case series reported survivorship of implants; in total, these articles report on 58 932 total hip replacements (range 38–22 036). 78 of 150 case series reported all-cause construct survival and 44 of these included confidence intervals (required for meta-analysis; figure 1). The table shows the characteristics of individual studies (appendix p 3 shows the full list of included articles and survival estimates).

**Table:** Characteristics of contributing data sources

	**Individual case-series articles**	**Australian Orthopaedic Association National Joint Replacement Registry Annual report 2017**	**Finnish Arthroplasty Report November 2017**
**Study level characteristics**			
Location	16 countries	Australia	Finland
Number of series included	44	36	56
Year of publication	1993–2017	2017	2017
**Participant level characteristics**			
Total joint replacements included	13 212	121 384	94 292
Mean age (years)	57·9*	67·7†	65–74‡
Proportion of female patients	56·2%§	55%†	58·5%†
Proportion of hips implanted for osteoarthritis	61·8%§	88·5%†	Not reported

* Weighted mean for age by number in study.

† All conventional total hip replacements in report.

‡ Exact figure not reported; the median is within this range.

§ Weighted proportion by number in study.

**Figure 2:**
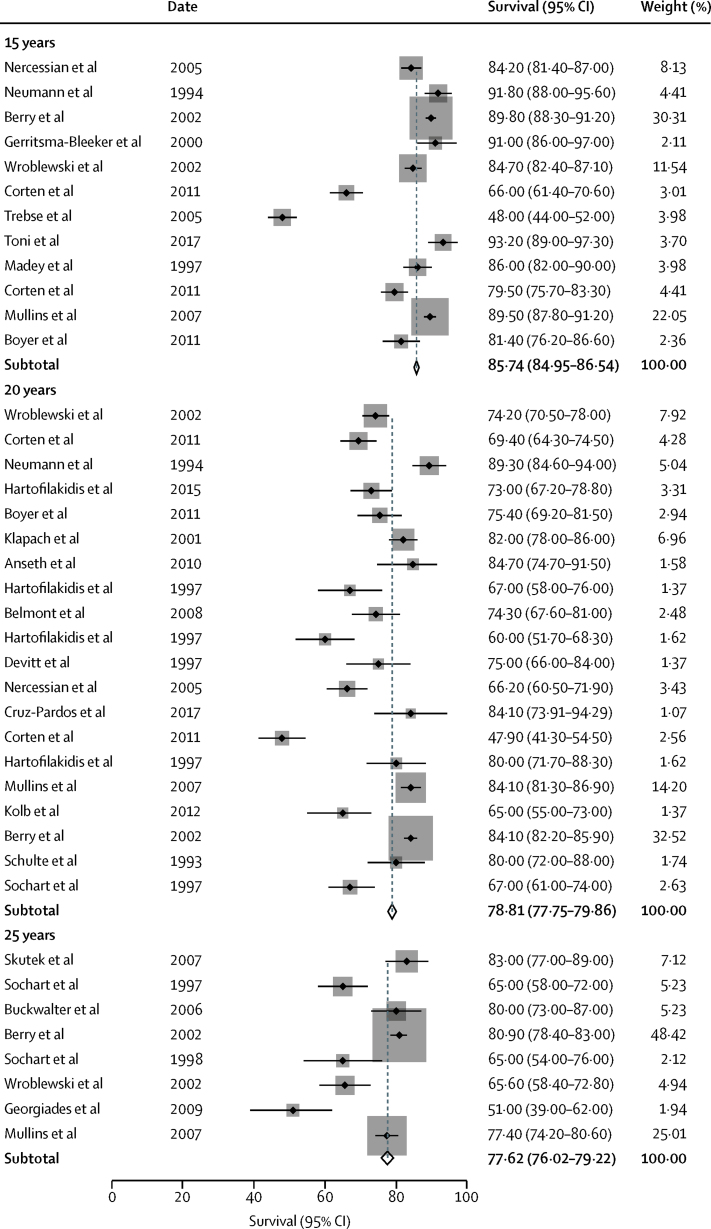
Estimates of survival at 15 years, 20 years, and 25 years from case series

The 44 case series included reported all-cause construct survival in 13 212 total hip replacements (range 73–2000), with follow-up ranging from 15 to 40 years. Not all series reported survival at exactly 15, 20, or 25 years and some series reported survival at more than one time. Pooled analysis of data derived from case series of total hip replacements reported at exactly 15 years, 20 years, and 25 years showed all-cause survivorship of the construct of 85·7% (95% CI 85·0–86·5) at 15 years, 78·8% (77·8–79·9) at 20 years, and 77·6% (76·0–79·2) at 25 years (figure 2).

In analysis in which survival at timepoints that were not exactly 15, 20, or 25 years were rounded down to the closest point (to include as many series as possible in our analyses), which gave pooled survival of 87·9% (87·2–88·5) at 15 years, 78·9% (77·9–80·0) at 20 years, and 76·6% (75·1–78·2) at 25 years (appendix p 8).

Generally, the quality of published case series was low. The quality assessment showed that 24 (54·5%) of 44 series were consecutive, none were multicentre, five (11·4%) had less than 20% follow-up, and seven (15·9%) used multivariable analyses.

**Figure 3:**
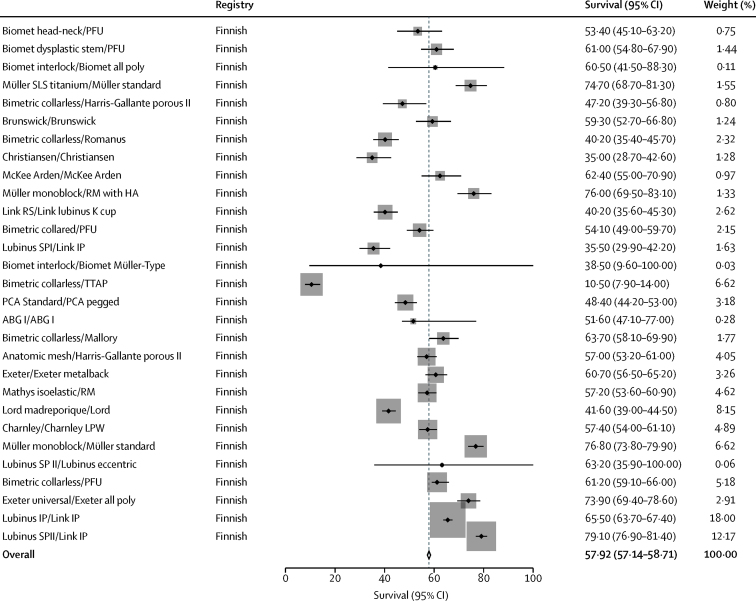
Estimates of survival at 25 years from registry annual reports

**Figure 4:**
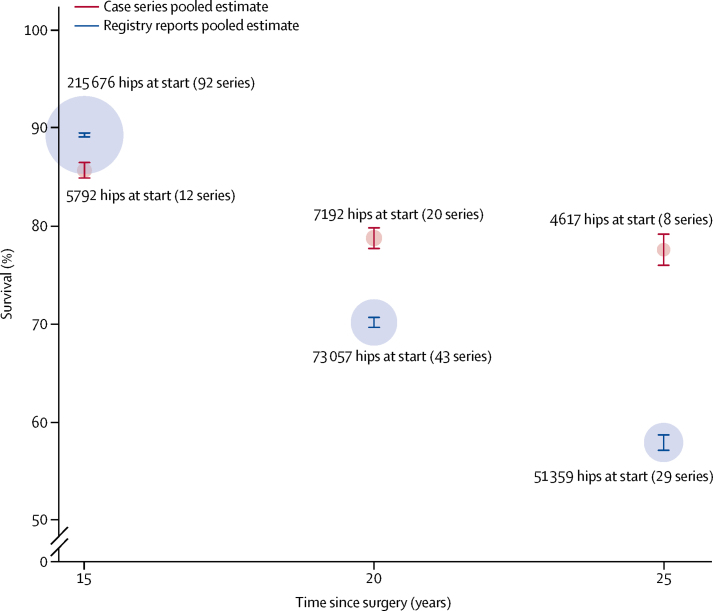
Comparison of pooled survival estimates from case series and registry reports at 15 years, 20 years, and 25 years The size of each circle is proportional to the total number of hip replacements at the start of all the series contributing to that pooled estimate.

The search of joint replacement registry reports yielded 92 series, and all provided confidence intervals. All 92 individual construct series reported survival analyses at 15 years (215 676 total hip replacements), 43 series at 20 years (73 057 total hip replacements), and 29 series at 25 years (51 359 total hip replacements). The pooled survival data derived from registry data showed all-cause construct survivorship of 89·4% (95% CI 89·2–89·6) at 15 years (appendix p 9), 70·2% (69·7–70·7) at 20 years (appendix p 10), and 57·9% (57·1–58·7) at 25 years (figure 3). The 15 year data included series originating from the Australian and Finnish registries, whereas the 20 year and 25 year data were exclusively from the Finnish registry. The other four arthroplasty registries with 15 years potential follow-up did not provide survival estimates broken down by the stem–cup combination and therefore could not be used in our analyses. Figure 4 shows a comparison of the point estimates at each timepoint for the data two sources.

## Discussion

Patients often ask clinicians how long their hip replacement will last. Until now, we have not had a generalisable answer to this question. We found that, according to registry data, just over half of hip replacements last 25 years. Published case series suggest better survivorship at 20 years and 25 years. The data from the two sources are similar at 15 years.

Concordance at 15 years is encouraging, but the differences at 20 and 25 years suggest that the two types of data probably have different sources of bias. Case series are particularly prone to selection bias, which is not a feature of registries and might lead to the study population not being representative of the target population. Case series are also more prone to publication bias, with clinicians and editors more likely to publicise results if good. Ideally, national registries capture the entire population, but in reality, they are limited by the quality of data submitted to the registry. Case series are usually selected and thus not necessarily generalisable, but if rigorously done can have better data completeness and contain a wider set of variables. The smaller numbers of patients in case series compared with registries means that loss to follow-up should be easier to prevent, whereas registry data are reliant on the correct linkage of a primary operation to the revision procedure. This might result in a form of selection bias in the registry data. In our review of case-series however, there was considerable variation in the number of patients lost to follow-up, suggesting that this is also a potential source of bias in case series. We think therefore that the difference in the survival estimates between the sources is in part down to the different biases in the two types of study. Regardless of potential sources of bias, the more conservative and generalisable estimates provided by the registry annual reports are likely to be more reliable.

The age and sex distribution of our studies are the same as those reported by the largest national registries such as the National Joint Registry for England, Wales, Northern Ireland and the Isle of Man (NJR), and the Swedish Hip Arthroplasty Register suggesting that our data are likely to be generalisable.^6^, ^7^ Survivorship of arthroplasties correlates strongly with age, sex, and implant selection and this must be considered when counselling patients.

Although our findings might be generalisable, they are also by definition historic, as there are secular changes in health service delivery, implant design, and patient characteristics. Several of the components that make up the hip replacement constructs in our analyses are no longer widely used. There were no articles reporting individual case series that assessed a construct used more than 250 times in the NJR 2016 annual report. In the 92 registry annual report series only four constructs (reported in five series) were still in common use and only 15-year survival data were available. Compared with our pooled results, these constructs had high 15-year survival (94·9%, 95% CI 93·9–94·8; appendix p 11). The constructs were the Exeter V40/Trident, Exeter V40/Exeter Contemporary Flanged, CPT/Trilogy, and CPT/ZCA. The 22 589 total hip replacements that used one of these combinations accounted for a quarter of total hip replacement constructs implanted in England and Wales in 2016.^6^ Using constructs with better survival should mean that revision rates will reduce in the future: overall revision rate by year of surgery has already decreased since 2008.^6^

Estimates of the lifetime risk of revision for hip replacements have been calculated using primary care databases (the UK Clinical Practice Research Datalink). Bayliss and colleagues reported a 20 year survival of 85·0% (95% CI 83·2–86·6) from analysis of 63 158 patients with a maximum follow-up of 20 years. This survival estimate is higher than our 20 year estimates. The mean age of patients in their cohort was 69 years and 62% were female, similar to our data. The difference in estimates could be due to several factors: the authors used a different database, covering a different time period (1991–2011 rather than 1980–2017 for the Finnish registry). Also, they used a life-table analysis method rather than the Kaplan-Meier estimator used in most studies included in our analysis. Most notable was the numbers of patients with hip replacements at each reporting time. Bayliss and colleagues reported 20 year survival data on 444 patients compared with the 73 057 hip replacements 20-year survival data in our study. The focus of Bayliss and colleagues' paper was the lifetime risk of revision of a joint replacement, which is not what we have reported. They surmised that the lifetime risk of revision changed according to the age of the patient at the time the hip replacement was implanted, with older patients having a lower lifetime risk of revision. Our lower survival at 20 years is likely to support their conclusion that if a patient has a hip replacement at a younger age, then the chances of the patient having revision surgery are higher. Several overall survival estimates have also been produced using data from Nordic arthroplasty registries. These studies mostly focus on specific cohorts, such as patients older than 55 years, younger than 55 years, or with a particular class of hip replacement (cemented or cementless), but two studies produced generalised estimates for all hip replacements. A 2014 collaborative report^28^ from the combined Nordic Arthroplasty Registry Association registry gave 15 year survival estimates of 86% (95% CI 85·7–86·9) in Denmark, 88% (87·6–88·3) in Sweden, 87% (86·4–87·4) in Norway, and 84% (82·9–84·1) in Finland. Fevang and colleagues' 2010 article^29^ reported a 20 year survival estimate of 77·3% (76·3–78·4) from the Norwegian registry with 280 hips at risk at this timepoint.

We found heterogeneity in the reporting of case series, particularly in the handling of patients lost to follow-up, the point at which survival was reported, and the number of hips at risk at the analysis point. We think that the primary endpoint of any analysis reporting joint replacement survivorship should be all-cause revision of any component of the construct. Although other endpoints, for example, failure of a single part of the construct or failure for a specific cause (such as aseptic loosening), were reported in series included in our review^30^, ^31^ and might be of interest, they should only be reported as secondary endpoints because they give a biased and unrealistic impression of survivorship.^32^ We have explained elsewhere,^33^ why the 1–Kaplan-Meier estimate is the most appropriate method of reporting survivorship (with a worst-case scenario for hips lost to follow-up) and should include the number of hip replacements at risk at the time of reporting.

Failure in arthroplasty can be measured many ways and patients who report failure in one metric often report success using another metric. Furthermore, revision surgery is a major intervention with uncertain outcome and thus both clinicians and patients might decide that the risks outweigh the benefits in individual cases. Failure measured by revision should thus be considered a best-case scenario. As the aim of surgery is to relieve pain, then a very stringent criterion could define failure as all patients with moderate-to-severe long-term pain. Beswick and colleagues^34^ have shown that 7–23% of patients who have not undergone revision would be regarded as failures using this outcome.

The main limitation of our pooled data is that they were not adjusted or stratified by patient factors that could have a role in determining survivorship. NJR data show that at 10 years, construct survivorship in men is roughly 97% for those aged older than 80 years, 96% for those aged 60–70 years, and 95% for those aged younger than 60 years. Women have slightly better construct survivorship at all ages than men. Without individual patient data, we have been unable to provide a risk estimate adjusted for individual characteristics such as age and sex, so our analyses provide an aggregated estimate for survival in all patients. Information was unavailable for the proportion of hips implanted for osteoarthritis in the Finnish Arthroplasty Registry. If this proportion was different to that seen in other countries, then it could be a source of selection bias. Poor implant choices such as metal-on-metal bearings will result in much higher failure rates.^35^, ^36^ Use of metal-on-metal bearing surfaces in the NJR peaked in 2008, with only 404 reported in the NJR in 2003.^36^ The hips included in our estimates were implanted before 2003, and thus should be unaffected by the higher failure rates of metal-on-metal hip replacements. Since 2003, the thresholds for undertaking surgery may have changed, which could alter future outcomes. As with all survival reports, we cannot account for a surgeon's willingness to revise a hip based on individual patient factors. This revision threshold could differ between countries and affect the generalisability of results between nations. Our pooled results from registries include data from only a few countries; however, the number of hip replacements was still far greater than that seen in individual case-series (215 676 *vs* 13 212).

Data contributing to 15-year survival came from both Australian and Finnish registries; however, results at 20 years and 25 years came only from the Finnish registry. We excluded papers not in English, which potentially excluded 13 further series. If all these case series were included they might have altered our pooled results, but we expect this effect would have been small. We assumed that survival estimates are equivalent to risks, for generating pooled estimates, and although the assumption that no censoring occurs (patients dying with a hip in situ) is clearly violated, it provides a useful method of aggregation in the absence of individual patient level data. The strengths of our study include an inclusive and comprehensive design, a-priori inclusion criteria, and a realistic interpretation of survivorship that accounts for all revisions and not a limited or biased subset.

In conclusion, although there is not enough information yet available to calculate exactly how long a hip replacement will last, using available arthroplasty registry data, we estimate that about three-quarters of hip replacements last 15–20 years and just over half of hip replacements last 25 years in patients with osteoarthritis (video).

## Supplementary Materials

Supplementary appendix

Supplementary video A summary of the findings from two systematic reviews and meta-analyses of national arthroplasty registry reports.
