# Supplementary material for: How long does a hip replacement last? A systematic review and meta-analysis of case series and national registry reports with more than 15 years of follow-up
Source: Lancet. 2019 Feb 16;393(10172):647–54. doi: 10.1016/S0140-6736(18)31665-9 (PMC6376618; doi:10.1016/S0140-6736(18)31665-9)

THE LANCET

Supplementary appendix

This appendix formed part of the original submission and has been peer reviewed. We post it as supplied by the authors.

Supplement to: Evans JT, Evans JP, Walker RW, Blom AW, Whitehouse MR, Sayers A. How long does a hip replacement last? A systematic review and meta-analysis of case series and national registry reports with more than 15 years of follow-up. *Lancet* 2019; **393:** 647–54.

Web Extra Material

Table 1: Search strategy formatted for search of Medline on Ovid Silver Platter

Hip replacement

Hip Prosthesis/ OR Arthroplasty, Replacement, Hip/ OR

(hip adj2 arthroplast$.mp) OR (hip adj2 replacement?.mp) OR

THA.mp OR THR.mp OR (TJR$.mp AND hip$.mp)

AND

Survival

Prosthesis Failure/ OR Survival Analysis/ OR

cox.mp OR proportional?hazard?.mp OR proportional hazard?.mp OR

cumulative?incidence?function.mp OR cumulative incidence function.mp OR CIF.mp OR

failure.mp OR

survival.mp OR survivor?ship.mp OR

revision?.mp OR

re?operation.mp OR re operation.mp OR

Kaplan?meier.mp OR Kaplan meier.mp OR KM.mp OR

product?limit?method.mp OR product limit method.mp

AND

Case-series

exp Cohort Studies/ OR

follow?up.mp OR follow up.mp OR series.mp OR cohort.mp OR

registry.mp OR registries.mp

AND

Long-term (minimum 15 years)

long?term.mp OR long term.mp OR

(1#* adj1 year?.mp) OR (2#* adj1 year?.mp) OR (3#* adj1 year?.mp) OR (4#* adj1 year?.mp)

Survival Adjacent to Long term (within 20 words) AND Hip replacement AND Case-series

Table 2: Reasons for exclusion at screening

Reason for exclusion	Number
Insufficient follow-up	897
Not an article regarding primary THR<br>e.g. revision/osteotomy	882
No survival analysis	435
Disease specific cohort other than osteoarthritis	320
Animal study	3
Solely paediatric study	2
n.b. some articles excluded for multiple reasons	

Table 3: Reasons for exclusion at review of full text

Reason for exclusion	Number
Insufficient follow-up/no survival analysis	121
Systematic review	22
Article based on registry data	20
Article not in English language	13
Unable to retrieve full text	3
Total	179

Table 4: Pooled estimates of survival for each available time point

Time (years)	Number of series	Total number of hips at start of series	Pooled survival estimate (%)	95% CI
15	12	5792	85·7	84·9-86·5
15·9	1	104	98·9	96·7-100
16	1	84	78·0	74·0-82·0
17	8	1327	88·5	87·0-90·1
18	2	159	80·5	74·9-86·0
18·8	1	105	87·0	82·6-91·4
19.8	1	93	79·7	70·0-88·0
20	20	7192	78·8	77·8-79·9
20·8	1	109	84·4	56·0-100
22	2	386	77·3	71·8-82·8
24·6	1	102	87·0	77·8-92·7
25	8	4617	77·6	76·0-79·2
27	2	1764	60·5	54·0-67·0
30	5	2103	62·2	58·4-66·1
35	2	423	71·7	65·0-78·3
40	1	2000	72·0	67·0-77·0

Table 5: Articles contributing to meta-analysis

Author	Year of publication	Time point (years post op)	Survival Estimate	Lower confidence interval	Upper confidence interval
Abdel, M. P.[1]	2016	40.0	72.0	67.0	77.0
Anseth, S. D.[2]	2010	20.0	84.7	74.7	91.5
Belmont, P.J.[3]	2008	20.0	74.3	67.6	81.0
Berry, D. J.[4]	2002	15.0	89.8	88.3	91.2
Berry, D. J.[4]	2002	20.0	84.1	82.2	85.9
Berry, D. J.[4]	2002	25.0	80.9	78.4	83.0
Boyer, B.[5]	2011	15.0	81.4	76.2	86.6
Boyer, B.[5]	2011	20.0	75.4	69.2	81.5
Boyer, B.[5]	2011	22.0	73.9	67.3	80.6
Buckwalter, A. E.[6]	2006	25	80	73	87
Busch, V. J.[7]	2012	17.0	89.0	85.0	93.0
Callaghan, J. J.[8]	2000	27.0	76.0	63.0	89.0
Callaghan, J.J..[9]	2009	35.0	78.0	70.0	86.0
Clarius, M.[10]	2010	17.0	75.0	65.0	85.0
Clarius, M.[11]	2010	17.0	49.0	41.0	57.0
Corten, K.[12]	2011	15.0	66.0	61.4	70.6
Corten, K.[12]	2011	20.0	47.9	41.3	54.5
Corten, K.[12]	2011	15.0	79.5	75.7	83.3
Corten, K.[12]	2011	20.0	69.4	64.3	74.5
Cruz-Pardos, A.[13]	2017	20	84.1	73.91	94.29
Devitt, A.[14]	1997	20.0	75.0	66.0	84.0
El Masri, F.[15]	2010	17.0	90.5	84.2	96.8
Georgiades, G.[16]	2009	25	51	39	62
Georgiades, G.[16]	2009	30	47	35	59
Gerritsma-Bleeker, C. L.[17]	2000	15.0	91.0	86.0	97.0
Gerritsma-Bleeker, C. L.[17]	2000	17.0	91.0	86.0	97.0
Gerritsma-Bleeker, C. L.[17]	2000	22.0	85.0	75.0	95.0
Hartofilakidis, G.[18]	1997	16.0	78.0	74.0	82.0
Hartofilakidis, G.[18]	1997	18.0	73.3	65.5	81.1
Hartofilakidis, G.[18]	1997	20.0	67.0	58.0	76.0
Hartofilakidis, G.[19]	1997	17	73	67.7	78.3
Hartofilakidis, G.[19]	1997	20	60	51.7	68.3
Hartofilakidis, G.[19]	1997	17	86	82.5	89.5
Hartofilakidis, G.[19]	1997	20	80	71.7	88.3
Hartofilakidis, G.[20]	2015	20.0	73.0	67.2	78.8
Hartofilakidis, G.[20]	2015	30.0	53.0	43.2	62.8
Ihle, M..[21]	2008	19.8	79.7	70.0	88.0
Kang, B. J.[22]	2015	15.9	98.9	96.7	100.0
Kawamura, H..[23]	2016	24.6	87.0	77.8	92.7
Keener, J. D.[24]	2003	30.0	60.0	53.0	67.0

Klapach, A.S.[25]	2001	20.0	82.0	78.0	86.0
Kolb, A..[26]	2012	20.0	65.0	55.0	73.0
Lass, R.[27]	2014	18.8	87.0	82.6	91.4
Madey, S.M..[28]	1997	15.0	86.0	82.0	90.0
Mullins, M. M.[29]	2007	15.0	89.5	87.8	91.2
Mullins, M. M.[29]	2007	20.0	84.1	81.3	86.9
Mullins, M. M.[29]	2007	25.0	77.4	74.2	80.6
Mullins, M. M.[29]	2007	30.0	73.3	67.2	79.4
Nercessian, O. A.[30]	2005	15.0	84.2	81.4	87.0
Nercessian, O. A.[30]	2005	20.0	66.2	60.5	71.9
Neumann, L.[31]	1994	15.0	91.8	88.0	95.6
Neumann, L.[31]	1994	20.0	89.3	84.6	94.0
Petsatodis, G. E.[32]	2010	20.8	84.4	56.0	100.0
Reigstad, O.[33]	2008	18.0	88.0	80.0	96.0
Schulte, K.R.[34]	1993	20.0	80.0	72.0	88.0
Skutek, M.[35]	2007	25.0	83.0	77.0	89.0
Sochart, D. H.[36]	1998	25.0	65.0	54.0	76.0
Sochart, D. H.[37]	1997	20	67	61	74
Sochart, D. H.[37]	1997	25	65	58	72
Suckel, A.[38]	2009	17.0	97.0	94.0	99.0
Toni, A.[39]	2017	15.0	93.2	89.0	97.3
Trebse, R.[40]	2005	15.0	48.0	44.0	52.0
Warth, L.C.[41]	2014	35.0	57.6	45.7	69.5
Wroblewski, B. M.[42]	2002	15.0	84.7	82.4	87.1
Wroblewski, B. M.[42]	2002	20.0	74.2	70.5	78.0
Wroblewski, B. M.[42]	2002	25.0	65.6	58.4	72.8
Wroblewski, B. M.[42]	2002	27.0	55.3	45.5	60.5
Wroblewski, B. M.[42]	2002	30.0	47.0	29.4	64.7

References

1. Abdel MP, Roth PV, Harmsen WS, Berry DJ. What is the lifetime risk of revision for patients undergoing total hip arthroplasty? a 40-year observational study of patients treated with the Charnley cemented total hip arthroplasty. *Bone Joint J* 2016; **98**(11): 1436-40.

2. Anseth SD, Pulido PA, Adelson WS, et al. Fifteen-year to twenty-year results of cementless Harris-Galante porous femoral and Harris-Galante porous I and II acetabular components. *J Arthroplasty* 2010; **25**(5): 687-91.

3. Belmont PJ, Jr., Powers CC, Beykirch SE, Hopper RH, Jr., Engh CA, Jr., Engh CA. Results of the anatomic medullary locking total hip arthroplasty at a minimum of twenty years. A concise follow-up of previous reports. *J Bone Joint Surg Am* 2008; **90**(7): 1524-30.

4. Berry DJ, Harmsen WS, Cabanela ME, Morrey BF. Twenty-five-year survivorship of two thousand consecutive primary Charnley total hip replacements: factors affecting survivorship of acetabular and femoral components. *J Bone Joint Surg Am* 2002; **84**(2): 171-7.

5. Boyer B, Philippot R, Geringer J, Farizon F. Primary total hip arthroplasty with dual mobility socket to prevent dislocation: a 22-year follow-up of 240 hips. *Inl Orthop* 2011: 1-8.

6. Buckwalter AE, Callaghan JJ, Liu SS, et al. Results of Charnley total hip arthroplasty with use of improved femoral cementing techniques. a concise follow-up, at a minimum of twenty-five years, of a previous report. *J Bone Joint Surg Am* 2006; **88**(7): 1481-5.

7. Busch VJ, Pouw MH, Laumen AM, van Susante JL, Vervest AM. Long-term outcome of 73 Zweymuller total hip prostheses with a screw cup in patients under 50 years of age. *Hip Int* 2012; **22**(3): 292-5.

8. Callaghan JJ, Albright JC, Goetz DD, Olejniczak JP, Johnston RC. Charnley total hip arthroplasty with cement. Minimum twenty-five-year follow-up. *J Bone Joint Surg Am* 2000; **82**(4): 487-97.

9. Callaghan JJ, Bracha P, Liu SS, Piyaworakhun S, Goetz DD, Johnston RC. Survivorship of a Charnley total hip arthroplasty. A concise follow-up, at a minimum of thirty-five years, of previous reports. *J Bone Joint Surg Am* 2009; **91**(11): 2617-21.

10. Clarius M, Jung AW, Raiss P, Streit MR, Merle C, Aldinger PR. Long-term results of the threaded Weill cup in primary total hip arthroplasty: a 15-20-year follow-up study. *Int Orthop* 2010; **34**(7): 943-8.

11. Clarius M, Jung AW, Streit MR, Merle C, Raiss P, Aldinger PR. Long-term results of the threaded Mecron cup in primary total hip arthroplasty : A 15-20-year follow-up study. *Int Orthop* 2010; **34**(8): 1093-8.

12. Corten K, Bourne RB, Charron KD, Au K, Rorabeck CH. What works best, a cemented or cementless primary total hip arthroplasty?: minimum 17-year followup of a randomized controlled trial. *Clin Orthop Relat Res* 2011; **469**(1): 209-17.

13. Cruz-Pardos A, Garcia-Rey E, Garcia-Cimbrelo E. Total Hip Arthroplasty with Use of the Cementless Zweymuller Alloclassic System: A Concise Follow-up, at a Minimum of 25 Years, of a Previous Report. *J Bone Joint Surg Am* 2017; **99**(22): 1927-31.

14. Devitt A, O'Sullivan T, Quinlan W. 16- to 25-year follow-up study of cemented arthroplasty of the hip in patients aged 50 years or younger. *J Arthroplasty* 1997; **12**(5): 479-89.

15. El Masri F, Kerboull L, Kerboull M, Courpied JP, Hamadouche M. Is the so-called 'French paradox' a reality?: long-term survival and migration of the Charnley-Kerboull stem cemented line-to-line. *J Bone Joint Surg Br* 2010; **92**(3): 342-8.

16. Georgiades G, Babis GC, Hartofilakidis G. Charnley low-friction arthroplasty in young patients with osteoarthritis: outcomes at a minimum of twenty-two years. *J Bone Joint Surg Am* 2009; **91**(12): 2846-51.

17. Gerritsma-Bleeker CL, Deutman R, Mulder TJ, Steinberg JD. The Stanmore total hip replacement. A 22-year follow-up. *J Bone Joint Surg Br* 2000; **82**(1): 97-102.

18. Hartofilakidis G, Karachalios T, Zacharakis N. Charnley low friction arthroplasty in young patients with osteoarthritis. A 12- to 24-year clinical and radiographic followup study of 84 cases. *Clin Orthop Relat Res* 1997; (341): 51-4.

19. Hartofilakidis G. Survival of the Charnley low-friction arthroplasty. A 12-24-year follow-up of 276 cases. *Acta Orthop Scand Suppl* 1997; **275**: 27-9.

20. Hartofilakidis GC, Lampropoulou-Adamidou KI, Stathopoulos IP, Vlamis JA. The Outcome of 241 Charnley Total Hip Arthroplasties Performed by One Surgeon 30 to 40 Years Ago. *J Arthroplasty* 2015; **30**(10): 1767-71.

21. Ihle M, Mai S, Pfluger D, Siebert W. The results of the titanium-coated RM acetabular component at 20 years: a long-term follow-up of an uncemented primary total hip replacement. *J Bone Joint Surg Br* 2008; **90**(10): 1284-90.

22. Kang BJ, Ha YC, Ham DW, Hwang SC, Lee YK, Koo KH. Third-generation alumina-on-alumina total hip arthroplasty: 14 to 16-year follow-up study. *J Arthroplasty* 2015; **30**(3): 411-5.

23. Kawamura H, Mishima H, Sugaya H, Nishino T, Shimizu Y, Miyakawa S. The 21- to 27-year results of the Harris-Galante cementless total hip arthroplasty. *J Orthop Sci* 2016; **21**(3): 342-7.

24. Keener JD, Callaghan JJ, Goetz DD, Pederson DR, Sullivan PM, Johnston RC. Twenty-five-year results after Charnley total hip arthroplasty in patients less than fifty years old: a concise follow-up of a previous report. *J Bone Joint Surg Am* 2003; **85-A**(6): 1066-72.

25. Klapach AS, Callaghan JJ, Goetz DD, Olejniczak JP, Johnston RC. Charnley total hip arthroplasty with use of improved cementing techniques: a minimum twenty-year follow-up study. *J Bone Joint Surg Am* 2001; **83-A**(12): 1840-8.

26. Kolb A, Grubl A, Schneckener CD, et al. Cementless total hip arthroplasty with the rectangular titanium Zweymuller stem: a concise follow-up, at a minimum of twenty years, of previous reports. *J Bone Joint Surg Am* 2012; **94**(18): 1681-4.

27. Lass R, Grubl A, Kolb A, et al. Primary cementless total hip arthroplasty with second-generation metal-on-metal bearings: a concise follow-up, at a minimum of seventeen years, of a previous report. *J Bone Joint Surg Am* 2014; **96**(5): e37.

28. Madey SM, Callaghan JJ, Olejniczak JP, Goetz DD, Johnston RC. Charnley total hip arthroplasty with use of improved techniques of cementing. The results after a minimum of fifteen years of follow-up. *J Bone Joint Surg Am* 1997; **79**(1): 53-64.

29. Mullins MM, Norbury W, Dowell JK, Heywood-Waddington M. Thirty-year results of a prospective study of Charnley total hip arthroplasty by the posterior approach. *J Arthroplasty* 2007; **22**(6): 833-9.

30. Nercessian OA, Martin G, Joshi RP, Su BW, Eftekhar NS. A 15- to 25- year follow-up study of primary Charnley low-friction arthroplasty: a single surgeon series. *J Arthroplasty* 2005; **20**(2): 162-7.

31. Neumann L, Freund KG, Sorenson KH. Long-term results of Charnley total hip replacement. Review of 92 patients at 15 to 20 years. *J Bone Joint Surgery Br* 1994; **76**(2): 245-51.

32. Petsatodis GE, Papadopoulos PP, Papavasiliou KA, Hatzokos IG, Agathangelidis FG, Christodoulou AG. Primary cementless total hip arthroplasty with an alumina ceramic-on-ceramic bearing: results after a minimum of twenty years of follow-up. *J Bone Joint Surg Am* 2010; **92**(3): 639-44.

33. Reigstad O, Siewers P, Rokkum M, Espehaug B. Excellent long-term survival of an uncemented press-fit stem and screw cup in young patients: follow-up of 75 hips for 15-18 years. *Acta Orthop* 2008; **79**(2): 194-202.

34. Schulte KR, Callaghan JJ, Kelley SS, Johnston RC. The Outcome of Charnley Total Hip-Arthroplasty with Cement after a Minimum 20-Year Follow-Up. *J Bone Joint Surg Am* 1993; **75a**(7): 961-75.

35. Skutek M, Bourne RB, Rorabeck CH, Burns A, Kearns S, Krishna G. The twenty to twenty-five-year outcomes of the Harris design-2 matte-finished cemented total hip replacement. A concise follow-up of a previous report. *J Bone Joint Surg Am* 2007; **89**(4): 814-8.

36. Sochart DH, Porter ML. Long-term results of cemented Charnley low-friction arthroplasty in patients aged less than 30 years. *J Arthroplasty* 1998; **13**(2): 123-31.
37. Sochart DH, Porter ML. The long-term results of Charnley low-friction arthroplasty in young patients who have congenital dislocation, degenerative osteoarthrosis, or rheumatoid arthritis. *J Bone Joint Surg Am* 1997; **79**(11): 1599-617.
38. Suckel A, Geiger F, Kinzl L, Wulker N, Garbrecht M. Long-term results for the uncemented Zweymuller/Alloclassic hip endoprosthesis. A 15-year minimum follow-up of 320 hip operations. *J Arthroplasty* 2009; **24**(6): 846-53.
39. Toni A, Giardina F, Guerra G, et al. 3rd generation alumina-on-alumina in modular hip prosthesis: 13 to 18 years follow-up results. *Hip Int* 2017; **27**(1): 8-13.
40. Trebse R, Milosev I, Kovac S, Mikek M, Pisot V. Poor results from the isoelastic total hip replacement: 14-17-year follow-up of 149 cementless prostheses. *Acta Orthop* 2005; **76**(2): 169-76.
41. Warth LC, Callaghan JJ, Liu SS, Klaassen AL, Goetz DD, Johnston RC. Thirty-five-year results after Charnley total hip arthroplasty in patients less than fifty years old. A concise follow-up of previous reports. *J Bone Joint Surg Am* 2014; **96**(21): 1814-9.
42. Wroblewski BM, Siney PD, Fleming PA. Charnley low-frictional torque arthroplasty in patients under the age of 51 years. Follow-up to 33 years. *J Bone Joint Surg Br* 2002; **84**(4): 540-3.

Figure 1: Forest plot of estimates for reported survival from case-series with reporting time rounded down to nearest 5 year interval

Author	Date	Series reporting time (years)	Survival (95% CI)	% Weight
**15-20 Years**				
Wroblewski, B. M.	2002	15	84.70 (82.40, 87.10)	7.19
Gerritsma-Bleeker, C. L.	2000	15	91.00 (86.00, 97.00)	1.31
Nercessian, O. A.	2005	15	84.20 (81.40, 87.00)	5.06
Corten, K.	2011	15	66.00 (61.40, 70.60)	1.88
Neumann, L.	1994	15	91.80 (88.00, 95.60)	2.75
Corten, K.	2011	15	79.50 (75.70, 83.30)	2.75
Madey, S.M.	1997	15	86.00 (82.00, 90.00)	2.48
Berry, D. J.	2002	15	89.80 (88.30, 91.20)	18.88
Trebse, R.	2005	15	48.00 (44.00, 52.00)	2.48
Boyer, B.	2011	15	81.40 (76.20, 86.60)	1.47
Mullins, M. M.	2007	15	89.50 (87.80, 91.20)	13.74
Toni, A.	2017	15	93.20 (89.00, 97.30)	2.31
Kang, B. J.	2015	15.9	98.90 (96.70, 100.00)	14.58
Hartofilakidis, G.	1997	16	78.00 (74.00, 82.00)	2.48
Gerritsma-Bleeker, C. L.	2000	17	91.00 (86.00, 97.00)	1.31
Suckel, A.	2009	17	97.00 (94.00, 99.00)	6.35
Hartofilakidis, G.	1997	17	86.00 (82.50, 89.50)	3.24
Clarius, M.	2010	17	49.00 (41.00, 57.00)	0.62
Hartofilakidis, G.	1997	17	73.00 (67.70, 78.30)	1.41
El Masri, F.	2010	17	90.50 (84.20, 96.80)	1.00
Busch, V. J.	2012	17	89.00 (85.00, 93.00)	2.48
Clarius, M.	2010	17	75.00 (65.00, 85.00)	0.40
Reigstad, O.	2008	18	88.00 (80.00, 96.00)	0.62
Hartofilakidis, G.	1997	18	73.30 (65.50, 81.10)	0.65
Lass, R.	2014	18.8	87.00 (82.60, 91.40)	2.05
Ihle, M.	2008	19.8	79.70 (70.00, 88.00)	0.49
Subtotal			87.87 (87.24, 88.50)	100.00
**20-25 Years**				
Cruz-Pardos, A.	2017	20	84.10 (73.91, 94.29)	1.01
Hartofilakidis, G.	1997	20	80.00 (71.70, 88.30)	1.53
Kolb, A.	2012	20	65.00 (55.00, 73.00)	1.30
Hartofilakidis, G.	2015	20	73.00 (67.20, 78.80)	3.13
Anseth, S. D.	2010	20	84.70 (74.70, 91.50)	1.49
Belmont, P.J.	2008	20	74.30 (67.60, 81.00)	2.34
Corten, K.	2011	20	69.40 (64.30, 74.50)	4.04
Devitt, A.	1997	20	75.00 (66.00, 84.00)	1.30
Mullins, M. M.	2007	20	84.10 (81.30, 86.90)	13.41
Sochart, D. H.	1997	20	67.00 (61.00, 74.00)	2.49
Neumann, L.	1994	20	89.30 (84.60, 94.00)	4.76
Corten, K.	2011	20	47.90 (41.30, 54.50)	2.41
Boyer, B.	2011	20	75.40 (69.20, 81.50)	2.78
Wroblewski, B. M.	2002	20	74.20 (70.50, 78.00)	7.48
Hartofilakidis, G.	1997	20	67.00 (58.00, 76.00)	1.30
Nercessian, O. A.	2005	20	66.20 (60.50, 71.90)	3.24
Hartofilakidis, G.	1997	20	60.00 (51.70, 68.30)	1.53
Schulte	1993	20	80.00 (72.00, 88.00)	1.64
Klapach, A.S.	2001	20	82.00 (78.00, 86.00)	6.57
Berry, D. J.	2002	20	84.10 (82.20, 85.90)	30.72
Petsatodis, G. E.	2010	20.8	84.40 (56.00, 100.00)	0.22
Gerritsma-Bleeker, C. L.	2000	22	85.00 (75.00, 95.00)	1.05
Boyer, B.	2011	22	73.90 (67.30, 80.60)	2.38
Kawamura, H.	2016	24.6	87.00 (77.80, 92.70)	1.89
Subtotal			78.92 (77.90, 79.95)	100.00
**25-30 Years**				
Skutek	2007	25	83.00 (77.00, 89.00)	6.71
Sochart, D. H.	1997	25	65.00 (58.00, 72.00)	4.93
Mullins, M. M.	2007	25	77.40 (74.20, 80.60)	23.58
Georgiades, G.	2009	25	51.00 (39.00, 62.00)	1.83
Berry, D. J.	2002	25	80.90 (78.40, 83.00)	45.65
Wroblewski, B. M.	2002	25	65.60 (58.40, 72.80)	4.66
Sochart, D. H.	1998	25	65.00 (54.00, 76.00)	2.00
Buckwalter, A. E.	2006	25	80.00 (73.00, 87.00)	4.93
Callaghan, J. J.	2000	27	76.00 (63.00, 89.00)	1.43
Wroblewski, B. M.	2002	27	55.30 (45.50, 60.50)	4.29
Subtotal			76.64 (75.09, 78.20)	100.00

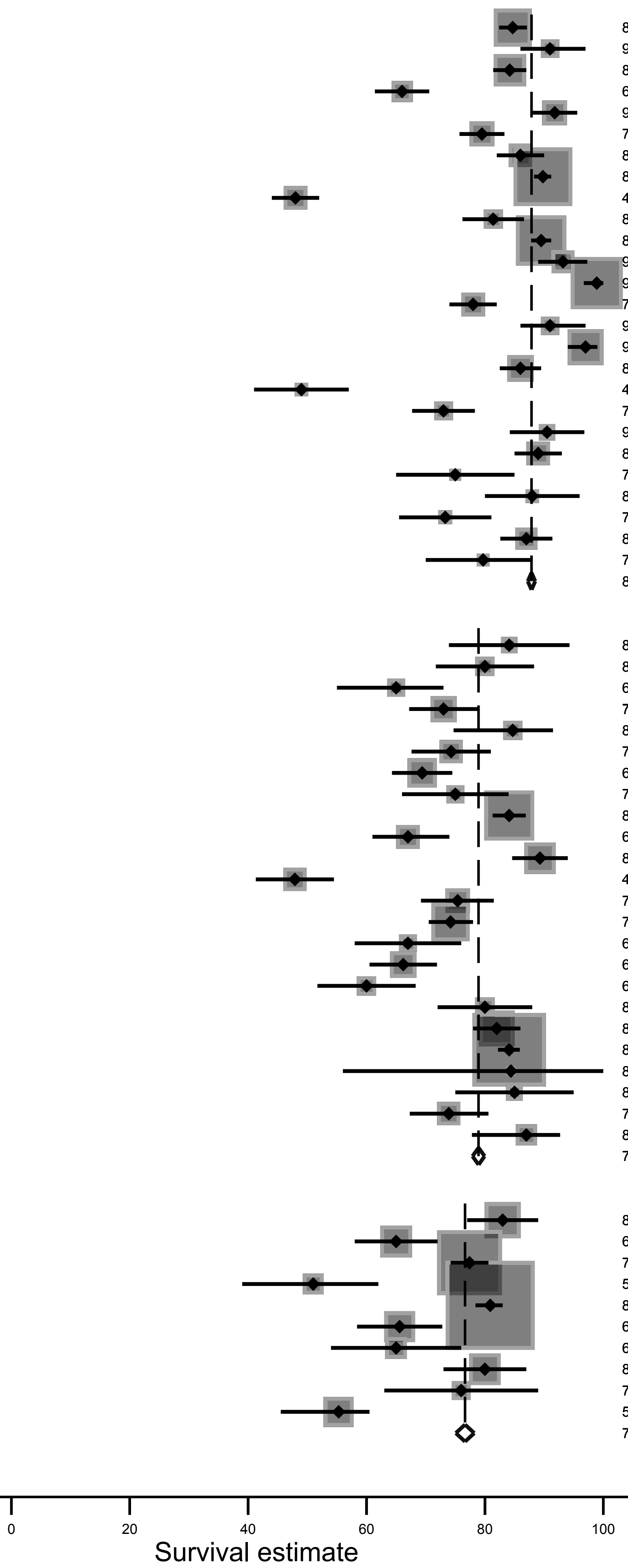

Figure 2: Forest plot of estimates for reported survival from registry annual reports at 15 years

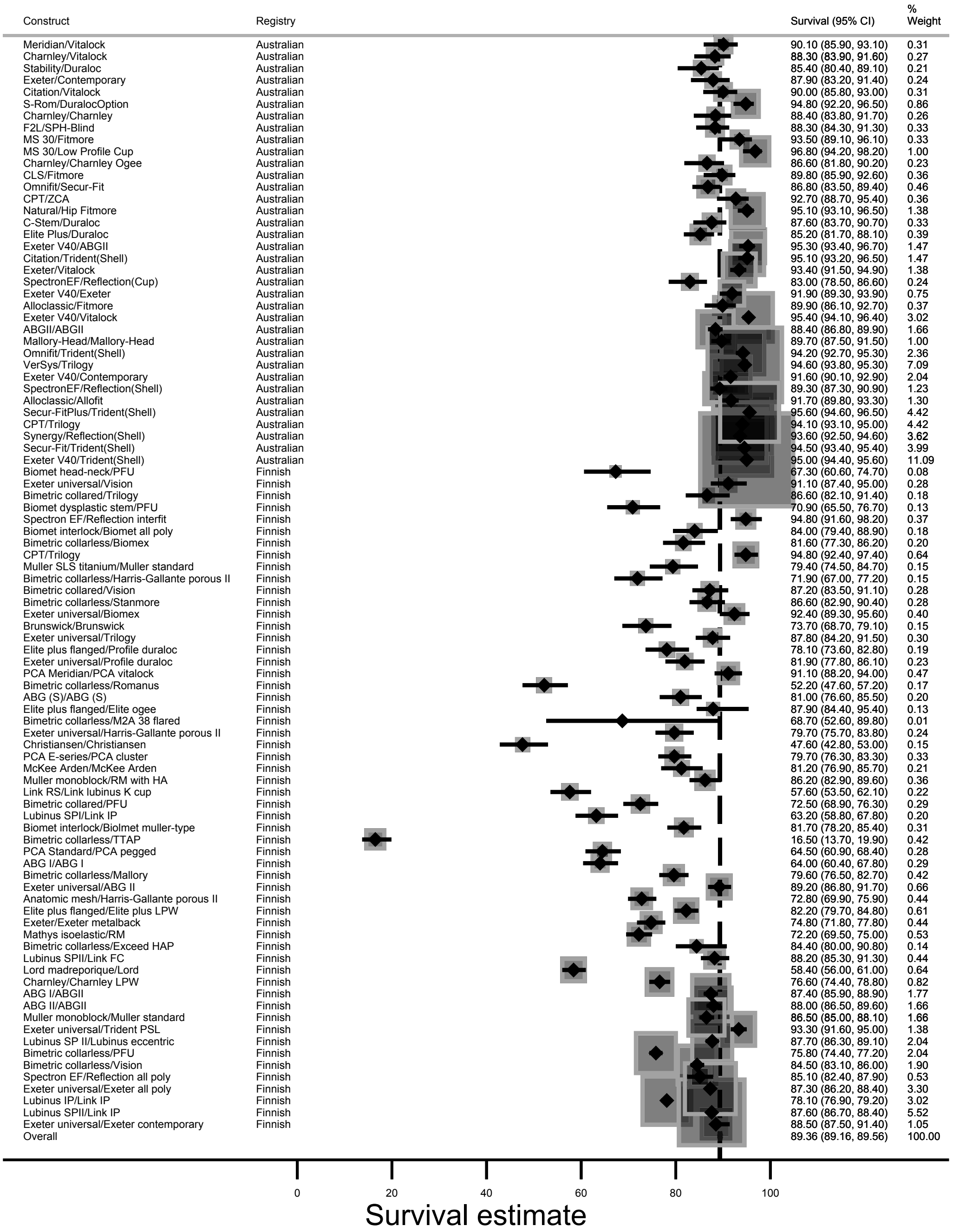

Figure 3: Forest plot of estimates for reported survival from registry annual reports at 20 years

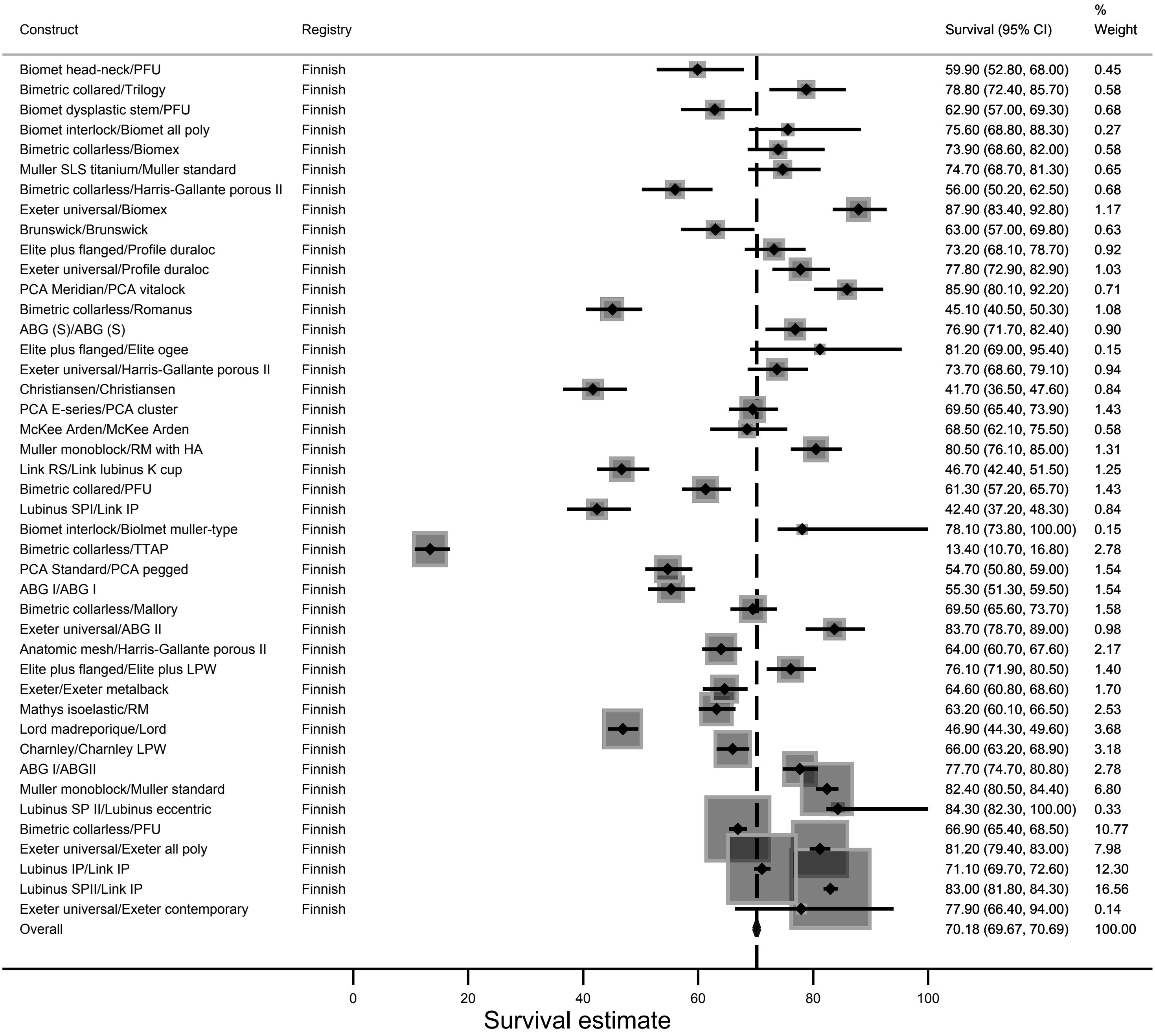

Figure 4: Forest plot of estimates for reported survival of constructs used more than 250 times in 2016 from registry annual reports at 15 years

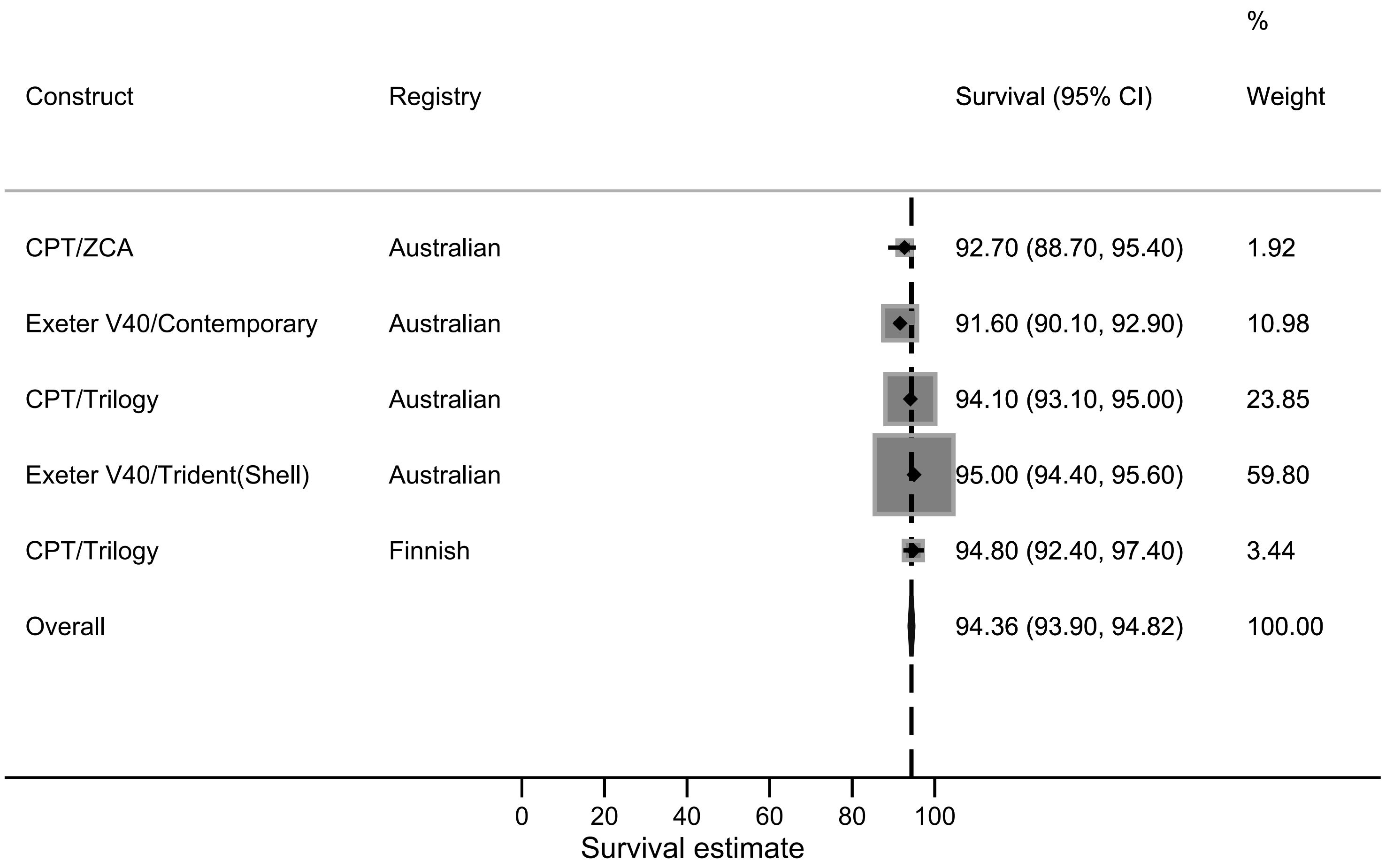

Supplement: Supplementary appendix [file mmc1.pdf]
